# Enhancement of cell migration and wound healing by nano-herb ointment formulated with biosurfactant, silver nanoparticles and *Tridax procumbens*

**DOI:** 10.3389/fmicb.2023.1225769

**Published:** 2023-08-02

**Authors:** Balakrishnan Muthukumar, M. S. Nandini, Punniyakotti Elumalai, Muthuraj Balakrishnan, Azhargarsamy Satheeshkumar, Mohamad S. AlSalhi, Sandhanasamy Devanesan, Punniyakotti Parthipan, Aruliah Rajasekar, Tabarak Malik

**Affiliations:** ^1^Environmental Molecular Microbiology Research Laboratory, Department of Biotechnology, Thiruvalluvar University, Serkkadu, Vellore, Tamil Nadu, India; ^2^Department of Microbiology, Sree Balaji Medical College and Hospital, Chennai, Tamil Nadu, India; ^3^Green Laboratory, Microbiology and Environmental Toxicology Laboratory, Saveetha Institute of Medical and Technical Sciences, Saveetha Dental College, Chennai, Tamilnadu, India; ^4^McKetta Department of Chemical Engineering, University of Texas at Austin, Austin, TX, United States; ^5^Department of Physics and Astronomy, College of Science, King Saud University, Riyadh, Saudi Arabia; ^6^Department of Biotechnology, Faculty of Science and Humanities, SRM Institute of Science and Technology, Kattankulathur, Chengalpattu, Tamil Nadu, India; ^7^Department of Biomedical Sciences, Institute of Health, Jimma University, Jimma, Ethiopia

**Keywords:** *Tridax procumbens*, biosurfactant, silver nanoparticle, herb ointment, cell migration

## Abstract

**Introduction:**

Ointments are generally used as a therapeutic agent for topical medication or transdermal drug delivery, such as wound healing and skin lesions.

**Methods:**

In this study, *Tridax procumbens* plant extract (0.7 g/mL) was used to prepare herbal-infused oil as the oil phase and gelatin-stabilized silver nanoparticle (G-AgNPs) (0.3 g/mL) as the aqueous phase. To blend the oil and aqueous phases, rhamnolipid biosurfactant with a critical micelle concentration of 55 mg/L from strain *Pseudomonas aeruginosa* PP4 has been used for herb ointment preparation. The average size of the synthesized G-AgNPs was observed between 10–30 nm and confirmed as spherical-shaped particles by TEM analysis. Subsequently, GC–MS and FTIR characterization are used to confirm herb ointment’s chemical and functional characteristics.

**Results:**

Based on the antibacterial studies, the highest microbial growth inhibition was observed for herb ointment, about 19.5 mm for the pathogen *Staphylococcus aureus* at the concentration of 100 μg/mL, whereas 15.5 mm was obtained for *Escherichia coli,* respectively. In addition, the minimum inhibitory concentration (MIC) assay showed negligible bacterial growth at 100 μg/mL for *S. aureus* and *E. coli,* respectively. Moreover, the cell viability assay for herb ointment exhibited low cytotoxic activity at higher concentrations (100 μg/mL) in Vero cell lines. In this study, wound scratch assay showed a significant cell migration rate (90 ± 2%) in 3 days of incubation than the control (62 ± 2%).

**Discussion:**

As a result, the biosurfactant-based nano-topical herb ointment revealed a low cytotoxic and higher cell migration capacity. Altogether, these findings highlighted the utility of this herb ointment in therapeutic applications such as wound healing.

## Introduction

The interruption of the cellular and anatomical continuity of the tissues that may result from physical, chemical, microbiological, and immunological harm is known as a wound (Rawat et al., 2012; Rajesh et al., 2020). Wound healing involves many biochemical events to repair and restore the damaged tissues through antioxidant defenses, epithelization, and remodeling phases (Cano Sanchez et al., 2018; Masson-Meyers et al., 2020). Several treatment options, including nonsteroidal, analgesics, commercial antibiotics, and anti-inflammatory drugs, are available for wound healing, but many of these therapies lead to undesirable side effects (Shenoy et al., 2011; Artym and Zimecki, 2023).

Ointments are semisolid and externally applied for skin treatments like burning, wounds, etc. (Thalkari et al., 2020; Laurent et al., 2023). Many commercial antibiotic ointments are used for dry and moist epidermal wounds on the skin; however, they can delay the healing process due to forming excessive bacterial growth on the burned/damaged skin layer (Kirsner and Eaglstein, 1993; Krysiak and Stachewicz, 2023). In addition, it may limit the usage of topical antibiotic ointments by causing an allergic reaction. Neomycin, Bacitracin, and Polymyxin are the topical antibiotic ointments used for wound healing treatments. Nevertheless, Neomycin and Bacitracin are the most severe allergic-causing antibiotic ointments identified by the North American Dermatitis researcher’s group (Prystowsky et al., 1979; Jennifer Ragi et al., 2011). In general, ointments are greasy or oily in consist of water in oil (W/O) emulsion (Agrawal et al., 2010; Chauhan and Gupta, 2020).

*Tridax procumbens* is an herbal plant from the *Asteraceae* family (Dattaray, 2022). The chemical compounds in the *T. procumbens* are flavonoid procumbenetin, sterols, polysaccharides, pentacyclic triterpenes, alkyl esters, and fatty acids (Gubbiveeranna and Nagaraju, 2016; Bhagat and Kondawar, 2019). The *T. procumbens* is also enriched with minerals such as sodium, zinc, copper, manganese, iron, and some other trace elements such as phosphorous, calcium, potassium, and magnesium (Oguntibeju, 2019). Several studies have reported that *T. procumbens* have been widely used in traditional medicine due to their antibacterial, antifungal, and anti-inflammatory activities. Besides, it has topical dermal wound healing properties against pathogens on the wound site (Kubendiran et al., 2021; Baile and Parmar, 2023).

Gelatin-stabilized silver nanoparticles (G-AgNPs) were used in this study as an aqueous phase to prepare nano-based herbal ointment. G-AgNPs generally have better antimicrobial properties against clinical pathogens (Chen et al., 2021; Singh and Mijakovic, 2022). Gelatin is one of the natural proteins which is used to prepare ointment and make cosmetics, capsules, and mainly for food processing (Abdullah et al., 2018; Khanh et al., 2019). In the literature, several studies revealed that silver nanoparticle (AgNPs) harms human cells because of their small particle size and can easily penetrate human tissues (Bagal-Kestwal et al., 2019). To avoid these problems, AgNPs have been prepared using gelatin as a stabilization agent, which could prevent direct contact with human cells and reduce the cytotoxicity level. An emulsifier was used as an additional compound to mix the immiscible solutions of two phases. Many ointments are available commercially, such as alkylphenol ethoxylates, lignin sulfonates, synthetic surfactants like sodium dodecyl sulfate (SDS), and others; however, the ionic charge of the used chemical surfactants could induce the cytotoxicity level in human cells (Tortella et al., 2020). To overcome these issues, biosurfactant was used as an emulsifier in this formulation. Generally, biosurfactants are low-toxic, eco-friendly, and readily biodegradable (Parthipan et al., 2021; Inès et al., 2023). Typically, biosurfactants are amphiphilic molecules with properties such as hydrophilic moiety, which comprises acids, peptides, and polysaccharides, whereas hydrophobic moiety includes saturated or unsaturated hydrocarbons and fatty acids. These structures give many functional properties to the biosurfactant, mainly capable of reducing the surface tension between liquid phases like water-in-oil or oil-in-water emulsion (Gautam and Tyagi, 2006; Jahan et al., 2020). Thus, A biosurfactant produced by *P. aeruginosa* was used as an efficient bio-emulsifier for the formulation of herb-oriented ointments.

Many traditional approaches deliver herb-oriented ointments, creams, gels, and lotions for several skin treatments (Chopra et al., 2023). However, these herb preparations showed poor penetration and limited bioavailability due to low hydrophobic and hydrophilic capacity, restricting their effectiveness (Sogut et al., 2021). On the other hand, nano-based approaches show significant results in wound management by enhancing transdermal penetration over traditional techniques (Barroso et al., 2020; Litany and Praseetha, 2022). This study used *T. procumbens* plant and G-AgNPs to prepare nano-based herb ointment due to their higher antimicrobial and wound healing properties. The rhamnolipid biosurfactant was used as emulsifying agent due to their better surface tension activity. This study aimed to explore the effectiveness of prepared biosurfactant-based nano-herbal ointment for the wound healing process. For the preparation of herb ointment, the herb-infused oil was designed as the oil phase using plant extract from *T. procumbens* and G-AgNPs as the aqueous phase. Further, their cytotoxicity and antibacterial activity have been performed against pathogenic bacterial strains.

## Materials and methods

### Chemicals and cell lines

The chemicals such as silver nitrate, gelatin, and Bushnell Haas Broth were obtained from Hi-media (Mumbai, India) and used as received. Dichloromethane, ethyl acetate, MTT [3-(4, 5-dimethylthiazol-2-yl)-2, 5-diphenyltetrazolium bromide], fetal bovine serum (FBS), dulbecco’s modified eagle medium (DMEM), streptomycin, penicillin and dimethyl sulfoxide (DMSO) were purchased from Sigma Aldrich. Vero cell line (African green monkey kidney normal cell line) was obtained from National Center for Cell Sciences (NCCS), Pune, India. The crude oil sample was collected from India’s Oil and natural gas corporation (ONGC) in Karaikal, India (Parthipan et al., 2017).

### Preparation of bacterial strain

The strain *P. aeruginosa* PP4 used in this study was isolated from oil-contaminated soil collected from the automobile industry at Sipcot, Vellore, Tamil Nadu, India. The NCBI number MZ855276 was received as the accession number for this strain. This strain was retrieved from glycerol stock stored at −20°C, and the purity of the strain was checked using the LB agar plate and subcultured using LB broth for further studies.

### Production and extraction of crude biosurfactant

The biosurfactant was produced from *P. aeruginosa* PP4, and the extraction procedure was followed as per our earlier publication (Muthukumar et al., 2022). In brief, the screening of biosurfactant production experiment was performed in a 500 mL Erlenmeyer flask containing 300 mL of sterile MSM (pH 7.0) supplement with filter sterilized crude oil (2%) as carbon and energy source. Further, bacterial culture (3%) was inoculated in the prepared growth medium and kept in a shaking incubator at 37°C for 7 days at 150 rpm under aerobic conditions. After 7 days of incubation, the medium was withdrawn from the flask and centrifuged at 10000 rpm to collect the cell-free supernatant and filtered using 0.2 μm filter paper to ensure the removal of complete bacterial cells. Then pH of the collected supernatant was adjusted to 2.0 using HCl (6 N) and kept at 4°C for overnight. The precipitated contents were centrifuged at 8000 rpm for 10 min to collect the crude biosurfactant. An equal volume of ethyl acetate was added into the collected supernatant in a separating funnel, mixed vigorously, and placed for phase separation for about 2 h without disruption. Subsequently, the biosurfactant was collected carefully from the organic layer, and extracted crude biosurfactant was dried using a vacuum oven. Then the obtained crude biosurfactant was further used as a bio-emulsifying agent for ointment preparation.

### Characterization of crude biosurfactant

The collected crude biosurfactant was characterized by thin-layer chromatography (TLC), and the detailed procedure was followed, as mentioned earlier (Wittgens et al., 2011; Parthipan et al., 2018). Briefly, the obtained biosurfactant was dissolved with 10 μL ethanol and spotted on a silica gel aluminium plate using the solvent system chloroform: methanol: acetic acid (65,15:2). The commercially obtained rhamnolipid sample solution (Sigma-Aldrich, India) (1 mg/mL) was used as standard. After this, the TLC plate was covered using 60% of H_2_SO_4_ (8.2 mL) in 42 mL of distilled water and orcinol (0.15 g) for stained the developed zones.

The obtained biosurfactant was further characterized by GCMS analysis and identified different chemical compounds. To confirm the functional constituents, it was subjected to FTIR analysis. The detailed procedures of GCMS and FTIR were followed as described by Parthipan et al. (2017).

### Collection and preparation of oil base from *Tridax procumbens*

The fresh *T. procumbens* plant leaves were collected from the Serkkadu village, Vellore, Tamil Nadu, India (13.02960^°^ S, 79.21102^°^ W). The collected leaves were washed with running tap water followed by double-deionized water to remove the dust particles and other waste debris. Then, the washed leaves were shade dried for three days, then in a hot air oven for 6 h at 50°C. After this, leaves were crushed as a powder using mortar and pestle. After that, obtained crude leaves extract of the powder (10 g) of *T. procumbens* was soaked with 100 mL of olive oil in a clean glass container and placed under sunlight for a week. The container was kept in a hot-air oven at 40°C at night to allow constant and gentle heat. After that, the oil was filtered with Whatman No.1 filter paper, and then the plant particles were removed using a 0.2 μm syringe filter (Kubendiran et al., 2021). Finally, obtained oil phase was used as herb-infused oil for herb ointment preparation. The obtained herb-infused oil (100 μL) was mixed with 2 mL of dichloromethane (DCM) for GCMS analysis. The prepared herb-infused oil was subjected to FTIR analysis to confirm the functional groups in the oil phase.

### Preparation of aqueous base

To prepare the aqueous phase, 0.01 g of gelatin was dissolved in double deionized water (10 mL) followed by 0.06 g of AgNO_3_ was added and mixed well; then, the solution was kept in the autoclave for 30 min at 121°C under 15 psi pressure. The formation of a clear yellow color solution was confirmed by the formation of G-AgNPs (Kubendiran et al., 2021; Lavanya et al., 2020). Further, this solution was used as an aqueous phase for the preparation of herb ointment. The prepared G-AgNPs particle size was characterized by using a UV–visible spectrophotometer (Model: Shimadzu UV-1800), and dynamic light scattering (DLS) was used for the confirmation of particle size using the Zeta Sizer-SZ 100 nano series, Horiba, Japan. Further, High-Resolution Transmission Electron Microscopy (HRTEM) (JEOL, Japan, JEM-2100 plus) has been used to measure synthesized nanoparticles’ size, shape, and appearance. In addition, EDAX analysis was used to confirm the elements present in the G-AgNPs.

### Preparation of ointment

To prepare the ointment, oil phase, aqueous phase, and biosurfactant as emulsifiers were used. Firstly, 30 and 70% of water in oil emulsion (W/O) was formulated as described by (Kubendiran et al., 2021). In brief, 0.7 g/mL of herb-infused oil was heated at 80°C for 5 min using a double boiler. The biosurfactant with a critical micelle concentration of 55 mg/L (with a surface tension of 42 ± 0.81 m/Nm) was added into the mild heated herb oil and stirred both solution mixtures slowly until dissolved. Simultaneously, 0.3 g/mL of colloidal silver (G-AgNPs) was heated at 80°C in a water bath. Then, the heated aqueous phase (G-AgNPs) was added to the oil phase and biosurfactant mixture. Then, the homogenous ointment mixture is obtained by constant stirring (Ferreira et al., 2017). Finally, the ointment was sealed with a sterile, airtight glass container for further experiments. The functional groups of ointment were confirmed by using FTIR.

### Antibacterial activity

The pure bacterial cultures namely, *Escherichia coli* (MTCC 1687) and *Staphylococcus aureus* (MTCC 737) were used for the antibacterial studies, which were obtained from the Institute of Microbial Technology (IMTECH), Chandigarh, India. Both bacterial strains were maintained using nutrient broth at 37°C (pH 7.5), and antibacterial activity was carried out in the presence of prepared infused herb oil, G-AgNPs, and herb ointment. The freshly prepared bacterial cultures were uniformly spread on the solidified Muller Hinton agar plates. Then, the well was carefully cut utilizing the cork borer, and finally, different concentrations (20–100 μg/mL) of herb-infused oil, G-AgNPs, and herb ointment were added. The plates were incubated for 24 h at 37°C for inhibition zone formation. Deionized water was monitored parallelly as a control. After 24 h of incubation, the zone formation was measured in mm (Hungund et al., 2015).

### Minimum inhibitory concentration

MIC test was carried out in test tubes. The freshly prepared bacterial cultures (*E. coli* 6.74× 10^7^ cell/mL and *S. aureus* – 6.98× 10^7^ cell/mL) were inoculated (10 μL) in each test tube containing 10 mL of nutrient broth and followed by the addition of different concentrations (20–100 μg/mL) of prepared ointment. A test tube containing nutrient broth without adding bacterial inoculum was maintained parallelly as a control. All the test tubes were incubated for 24 h at 37°C (250 rpm) in a shaking incubator. After 24 h of incubation, the least concentration of ointment showing no visible bacterial growth in the test tube was observed as MIC. The bacterial growth reduction was confirmed by a spectrophotometer at OD_600nm_ using Shimadzu UV-1800 UV (Krishnan et al., 2015; Kubendiran et al., 2021).

### Minimum bactericidal concentration

To observe the minimum bactericidal concentration (MBC) of two bacterial strains, the bacterial culture from each MIC test tube containing different concentrations (20–100 μg/mL) of ointment was streaked over the freshly prepared nutrient agar plates. Then all plates were incubated for 24 h at 37°C. After 24 h of incubation, no visible sign of growth was observed for the concentration was found to be MBC (Parvekar et al., 2020).

## Cytotoxic activity

### Cell culture maintenance

The obtained cells (Vero cell lines- African green monkey kidney normal cell line) were maintained by using Dulbecco’s modified eagle medium (DMEM) supplemented with 100 U/mL of penicillin, 100 μg/mL of streptomycin and 10% (v/v) heat-inactivated fetal bovine serum (FBS) in the logarithmic growth phase. Further, the cells were carefully maintained in the air-humidified incubator with a 5% CO_2_ atmosphere at 37°C (Tanaka et al., 1981; Castañeda et al., 2012).

### Cytotoxicity assay

The cytotoxicity test of the prepared herb ointment was examined against Vero cell lines by using MTT (3-[4, 5-dimethylthiazol-2-yl]-2, 5-diphenyltetrazolium bromide) assay as mentioned earlier (Mossman et al., 1983; Sumer et al., 2013; Srivastava et al., 2016). In brief, the cells were seeded carefully in the 96-well microplates (1 × 10^7^ cells/well), incubated in a CO_2_ incubator (5% CO_2_ atmosphere) for 48 h at 37°C, and allowed to grow about 70–80% confluence. After that, cells were treated with different concentrations like 20, 40, 60, 80, and 100 μg/mL of the sample and incubated for 24 h. After 24 h, the morphological changes were observed for both untreated (control) and treated cells using a digital inverted microscope (Magnus INVI, Noida) with 20X magnification and photographed the cells. Then the cells were washed using 20 μL of (MTT) solution (5 mg/mL in PBS) and phosphate-buffer saline (PBS- pH 7.4) and added into each well. Finally, plates were placed in the dark for 2 h at 37°C. Then the formazan crystals were further dissolved by adding 100 μL of DMSO solution, and the absorbance range was read spectrometrically using a multimode reader (Tecan, Austria) at 570 nm. The cell viability percentage (%) was calculated using the formula.

### Wound scratch assay

The wound healing assay was carried out as mentioned (Liang et al., 2007). This assay analyzed the prepared herb ointment against the Vero cell line. The 8 × 10^5^ cells/well were seeded in the 6-well tissue culture plates, and the middle confluence reached about 90% in the culture conditions. Then, the wound scratch was made using P10 tips, and the cell debris was removed carefully by washing twice with a fresh medium. Then, the created wound was exposed to 100 μL of prepared herb ointment, and the untreated cell was used as positive control parallelly. After, the plates were incubated for 72 h at 37°C with 5% of CO_2_ atmosphere. Then, the closure of the wound scratch and cell migration on the gap was observed using an inverted microscope (20X magnification), and the clear images were taken at 24 h intervals.

### Statistical analysis

All the tests were performed in triplicate times, and standard deviation values were provided for all the applicable tables and figures as ± SD. The variance was determined using ANOVA to appraise the *p* value. Graphpad Prism software was used to perform the statistical analysis, and Dunnett’s test determined the significant difference, and the values were considered when *p* < 0.05.

## Results and discussion

### Production and characterization of crude biosurfactant

The strain *P. aeruginosa* PP4 was used for biosurfactant production. As detailed in our earlier report, various biosurfactant screening methods confirmed this strain’s biosurfactant production ability (Muthukumar et al., 2022). The term “rhamnolipid” often refers to a kind of glycolipid biosurfactant that is generated from the *Pseudomonas* species (Abdel-Mawgoud et al., 2010; Khademolhosseini et al., 2019). Due to their non-toxicity, eco-friendly, and excellent surface and biological activities, this was widely applied for many sectors like environmental clean-up, food processing, cosmetics, pharmaceutical, and biomedicines (Gayathiri et al., 2022). Thus, we have chosen *P. aeruginosa* strain for therapeutic applications such as wound healing.

About 4.7 mg/L of crude biosurfactant was extracted after seven days of incubation and used as active surface molecules for further experiments. According to previous studies, (Parthipan et al., 2017), glycolipid biosurfactant contains a hydrophilic group of carbohydrates like glucose, rhamnose, mannose, sophorose, galactose, and hydrophobic chain of the fatty acid tail. Especially, rhamnolipid biosurfactant is one of the efficient biomolecules against microorganisms like bacteria, fungi, viruses, and mycoplasma because it plays a potential role in molecular mechanisms such as destabilizing the biological membranes through the generation of pores and ion channels (Thakur et al., 2020; Saikia et al., 2021). Moreover, the rhamnolipid biosurfactant could activate/inhibit some biological processes by modulating the various enzymes (Orive-Milla et al., 2020; Thakur et al., 2021). Besides, rhamnolipid biosurfactants have a remarkable ability to clear the interfacial tension between the liquid/liquid and solid/liquid phases and are applied in many biomedical applications like antitumor agents against human breast cancer cells, antimicrobial, antibiofilm activities and act as immunomodulators (Haeri et al., 2017; Mishra et al., 2021). As mentioned earlier, several studies have strongly determined that the biosurfactant produced by *P. aeruginosa* belongs to the rhamnolipids category, which contains a rhamnose-based head group and fatty acid tail group (Déziel et al., 1996).

The extracted crude biosurfactant was characterized by thin-layer chromatography (TLC), and the image is shown in Supplementary Figure S1 (Supplementary information). From this image, the developed spots of the produced biosurfactant from *P. aeruginosa* PP4 and the migration speed in lane 2 were almost similar with retention factor (R_f_ - 0.78) when compared to used standard rhamnolipid in lane 1, which indicates that the biosurfactant produced from *P. aeruginosa* PP4 is rhamnolipid in nature. Similar results were reported by Schenk et al. (1995) and Parthipan et al. (2018) that the *Rf* values of used biosurfactant were observed at about 0.74 and 0.73, respectively which confirmed the presence of rhamnolipid biosurfactant. Bhat et al. (2015) also reported that the retention factor (*Rf*) value of 0.73 was obtained and confirmed the presence of biosurfactant produced by the strain *P. aeruginosa* Pa24 was rhamnolipid.

**Figure 1:**
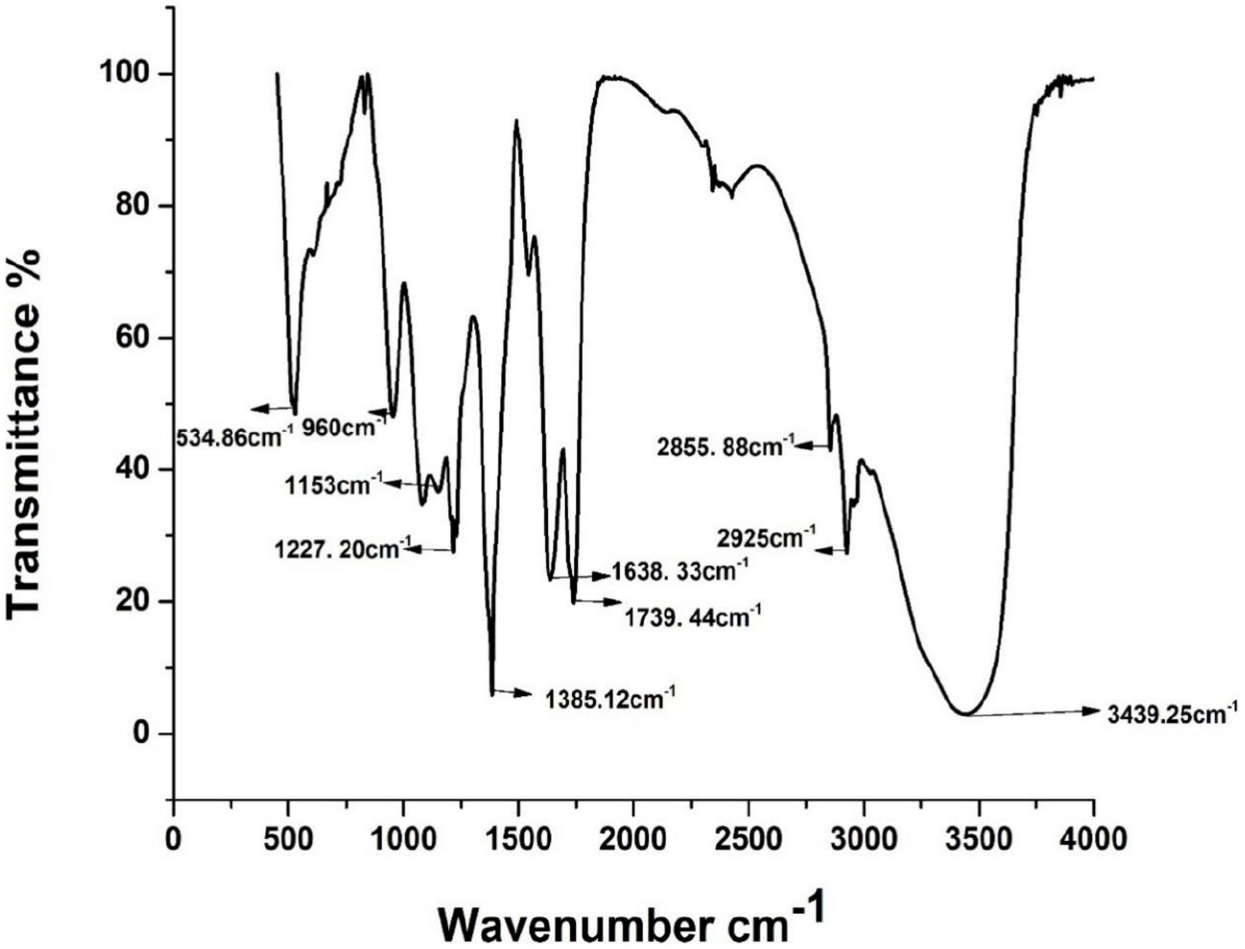
FTIR spectrum of extracted crude biosurfactant from *Pseudomonas aeruginosa* PP4.

The FTIR spectra of extracted biosurfactant are shown in Figure 1. As shown in Figure 1, the peak around 3,439 cm^−1^ indicates the presence of a-OH bond (Hydroxyl group). Absorption around 2,925 and 2,855 cm^−1^ confirmed the presence of the CH_3_ bond (methyl group) of aliphatic chains. The peaks around 1739 and 1,638 cm^−1^ represent the presence of carbonyl groups (-C=O). The band region at 1385 cm^−1^ corresponds to the COO- (antisymmetric stretching), and the C-H bending of-CH2 and CH_3_ groups was confirmed. The band around 1,079 cm^−1^ indicates the presence of carbohydrate compounds in the cyclic structure of the-COC- group. It reveals the presence of bonds between the carbon and hydroxyl groups in the chemical form of rhamnose rings. As reported by Bondarenko et al. (2010) and Jadhav et al. (2011), the results of FTIR profiles strongly confirmed that *P. aeruginosa* derived biosurfactant is the rhamnolipid. As a result, FTIR analysis confirmed that the isolated *P. aeruginosa* PP4 produced a glycolipid family of rhamnolipid biosurfactants.

**Figure 2:**
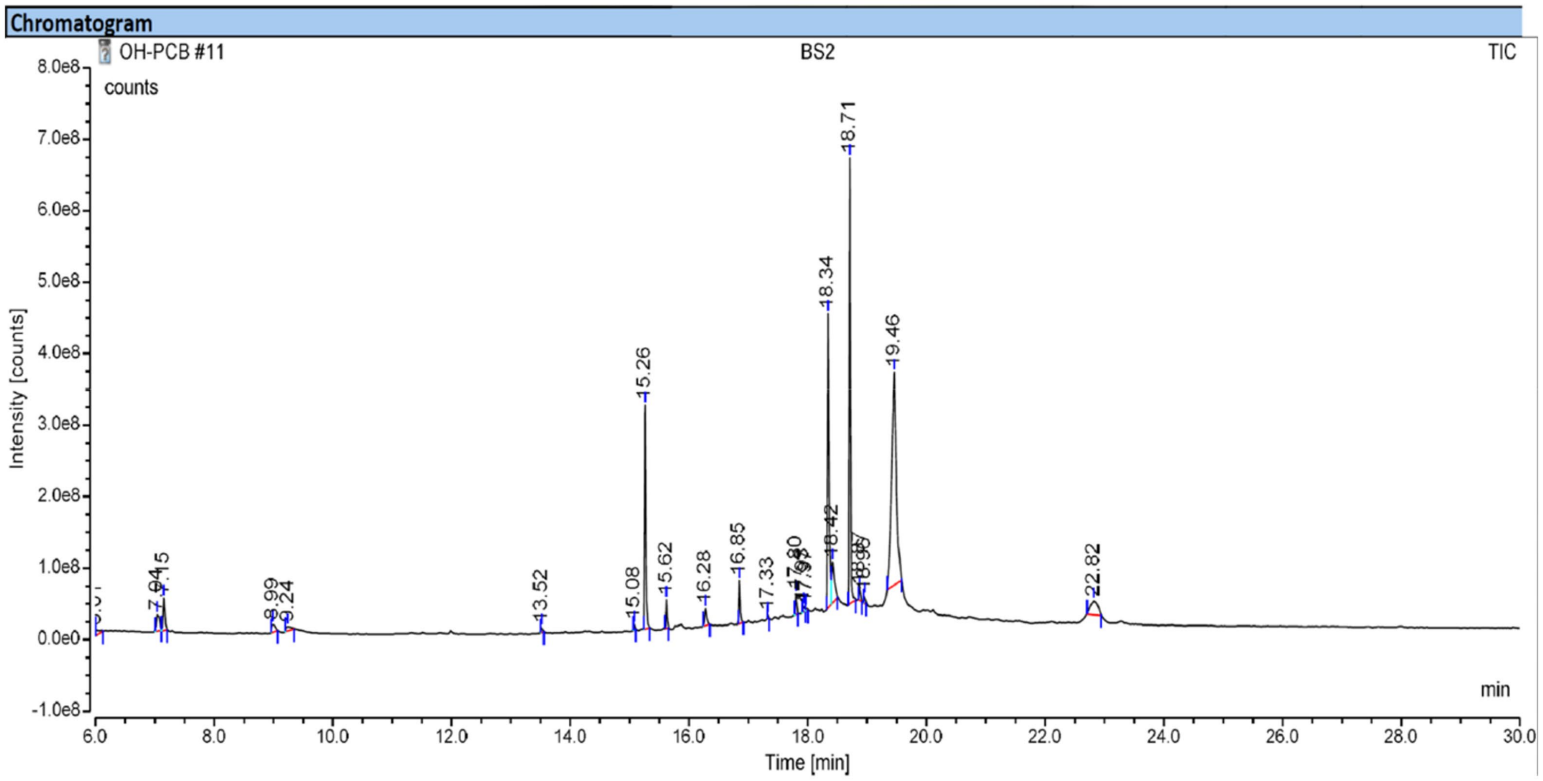
GCMS analysis of extracted crude biosurfactant from *Pseudomonas aeruginosa* PP4.

GCMS analysis was performed for extracted crude biosurfactants to identify the compounds in the biosurfactant. The GCMS chromatogram of the biosurfactant is shown in Figure 2. Various chemical compounds were identified that present in the produced biosurfactant at a different retention time of 13.51, 15.26, 16.84, 18.34, 18.71, and 19.45 from the standard library components as dodecanoic acid, dibutyl phthalate, hexadecenoic acid, 3-hydroxypropyl palmitate, phthalic acid and androst-7-ene,6,17-dione, respectively. Rath et al. (2016) reported the presence of dodecanoic acid and hexadecenoic acid in the rhamnolipid biosurfactant produced by *P. aeruginosa*. According to Rath et al. (2016), a similar result was obtained in this study, indicating that biosurfactant derived from *P. aeruginosa* was rhamnolipid in nature.

### Preparation and characterization of ointment

**SCHEME 1:**
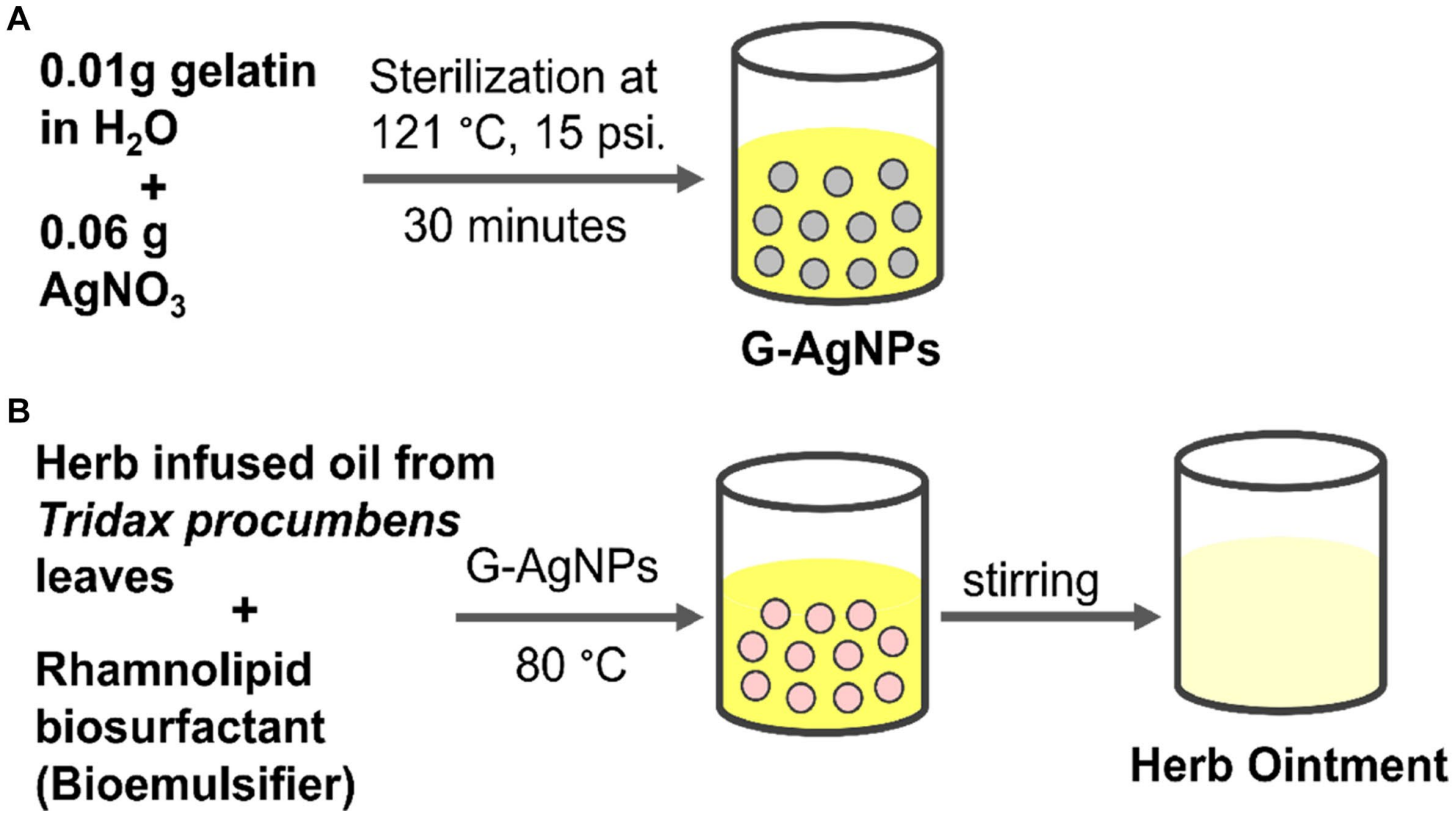
**(A)** Preparation of G-AgNPs; **(B)** Preparation of herb ointment using herb-infused oil from *Tridax procumbens* and biosurfactant.

The synthesis of G-AgNPs and preparation of herb ointment was shown in Scheme 1A,B. For the preparation of ointment, a higher quantity of oil phase was prepared over the aqueous phase to attain a waterproof nature and greasy level on the wound. Generally, biosurfactants produced by *Pseudomonas* species are used as an effective emulsifier than other synthetic surfactants (Ferreira et al., 2017; Kubendiran et al., 2021). Mostly used essential oil for clinical assays like wound healing, and the derived quantity of oil was less (Cimino et al., 2021). To overcome this issue and improve essential oil’s potential activity, herbal macerate procedures have been used to prepare the oil. In this study*, T. procumbens* plant leaves were soaked with olive oil for one week under sunlight. The extract of this plant leaves is effectively used for treating wound infections and showed efficient cytotoxic activity against tumor cells (Lavanya et al., 2020; Telange et al., 2022). According to previous studies, olive oil has higher emulsion activity, but soluble compounds like resins and essential oil can be dissolved when mild heat is applied (Wynn and Fougère, 2007).

**Figure 3:**
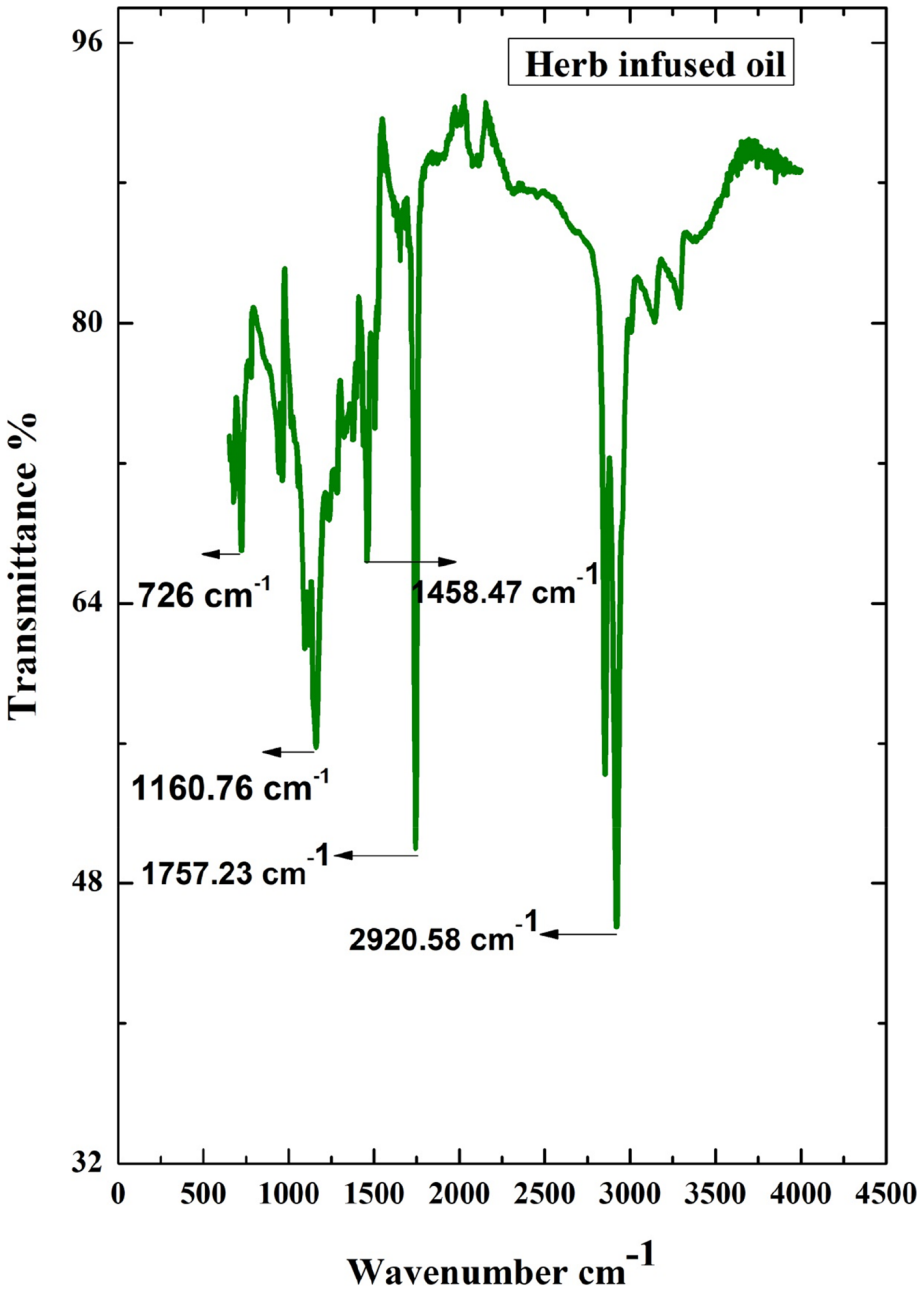
FTIR spectrum of prepared herbal infused oil from *Tridax procumbens.*

The FTIR spectra analysis of herb-infused oil is shown in Figure 3. Five major peaks were obtained from this spectrum, revealing the presence of different compounds in the herb-infused oil. The absorption peak at 2920 cm^−1^ indicates the-CH3 methyl group present in the infused oil. The band region around 1757 cm^−1^ corresponds to (-C=O) carbonyl stretching. The band at 1458 cm^−1^ confirmed the -C-H bending of the methylene group in the sample. The absorption peak around 1,160 cm^−1^ represents the C-O group of aliphatic ether. The presence of methyl group was confirmed by the peak region obtained at 726 cm^−1^. As a result, FTIR analysis confirmed that herb-infused oil contains different functional groups.

**Figure 4:**
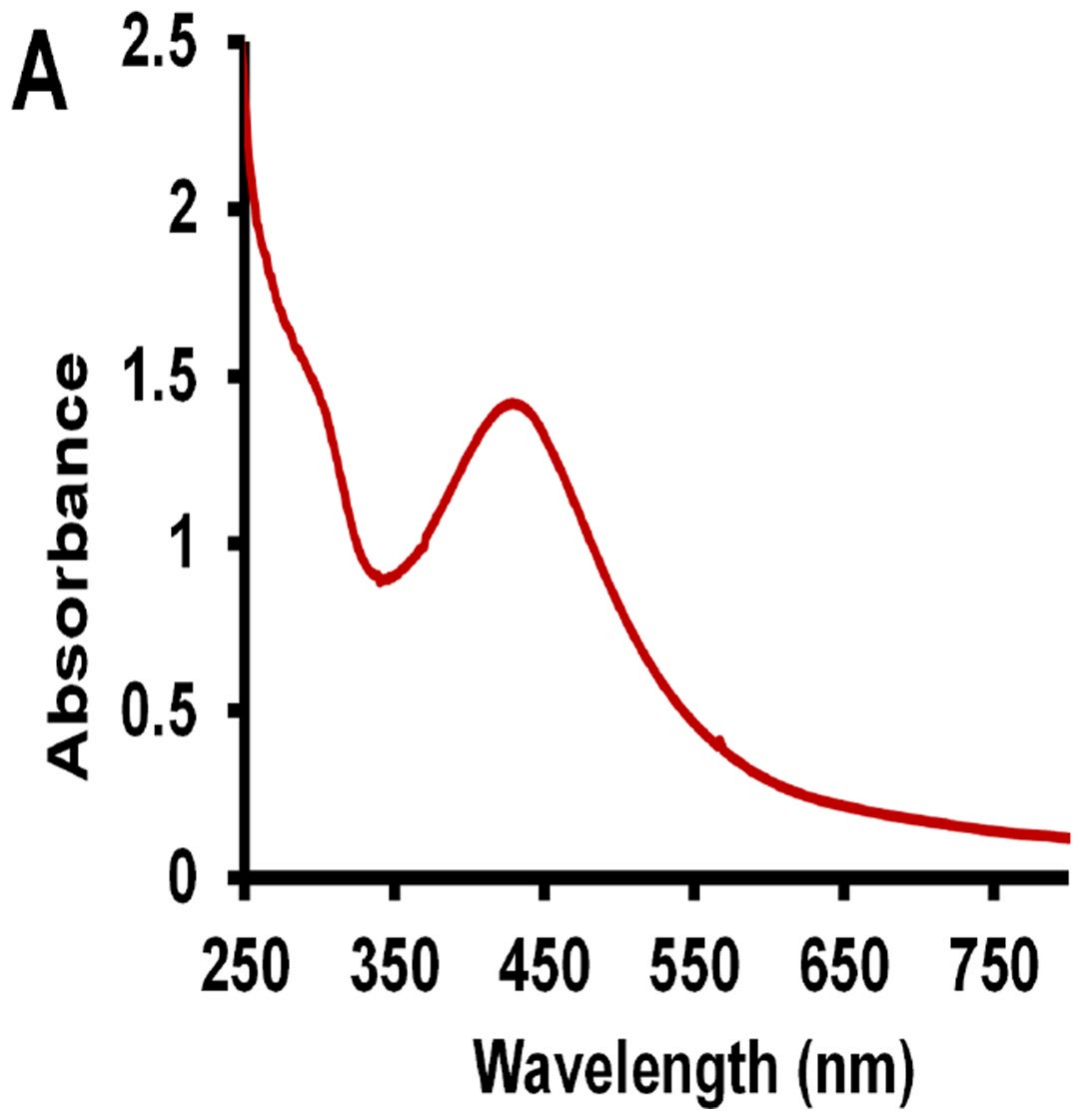
UV–Vis spectra of synthesized Gelatin stabilized silver nanoparticle (G-AgNPs).

The UV–vis spectroscopy was used to confirm the absorbance level of the G-AgNPs. Figure 4 shows the optical absorbance of freshly prepared G-AgNPs solution. The formation of AgNPs is demonstrated by the appearance of absorption peaks between 420–450 nm. Similar absorption peaks were obtained for AgNPs near 430 nm reported by Aravinthan et al. (2015). Thus, this result confirmed the heating of the silver nitrate solution with gelatin at autoclaving temperature leading to the reduction of G-AgNPs at the nanoscale.

**Figure 5:**
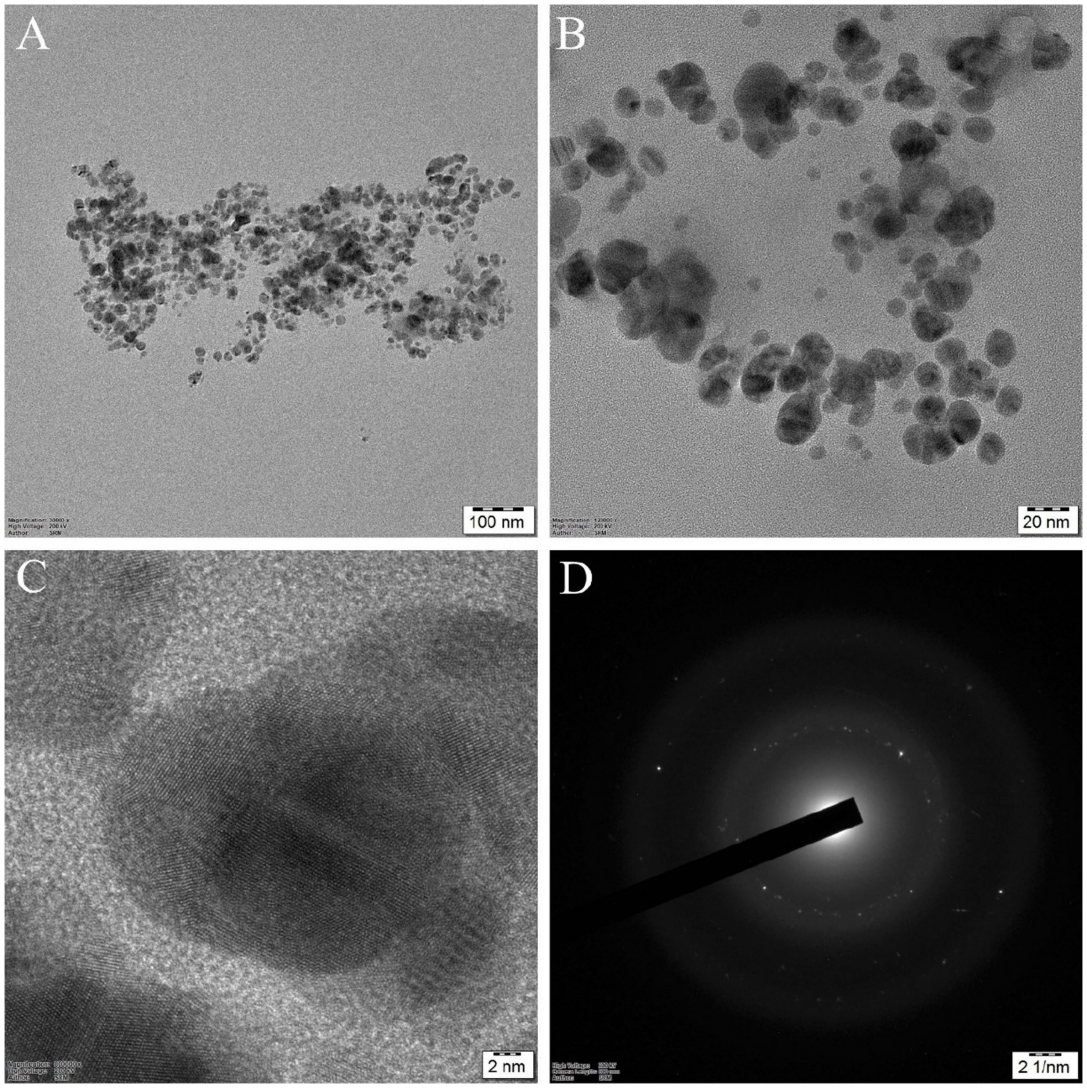
HR-TEM images of silver nanoparticles: **(A)** and **(B)** G-AgNPs at different magnifications; **(C)** High-resolution image of silver nanoparticles with clear lattice fringes shows (111) crystallographic planes; **(D)** SAED pattern of G-AgNPs with crystalline structure.

Transmission electron microscopy (TEM) indicates the size and morphology of the synthesized G-AgNPs, as shown in Figure 5. Different scales of magnifications were presented in Figures 5A,B. From these images, it was apparent that the nanomaterials were uniform in size and shape and obtained without agglomeration. Spherical-shaped nanoparticles with 10–30 nm in the ranges were obtained. Further, the clear lattice fringes were observed with the solid crystalline structure (Figure 5C). In the end, a selective area electron diffraction (SAED) pattern was observed, which was obtained according to the XRD diffraction pattern. The obtained nanoparticles were extremely nanoscale compared to previous reports (Aravinthan et al., 2015).

**Figure 6:**
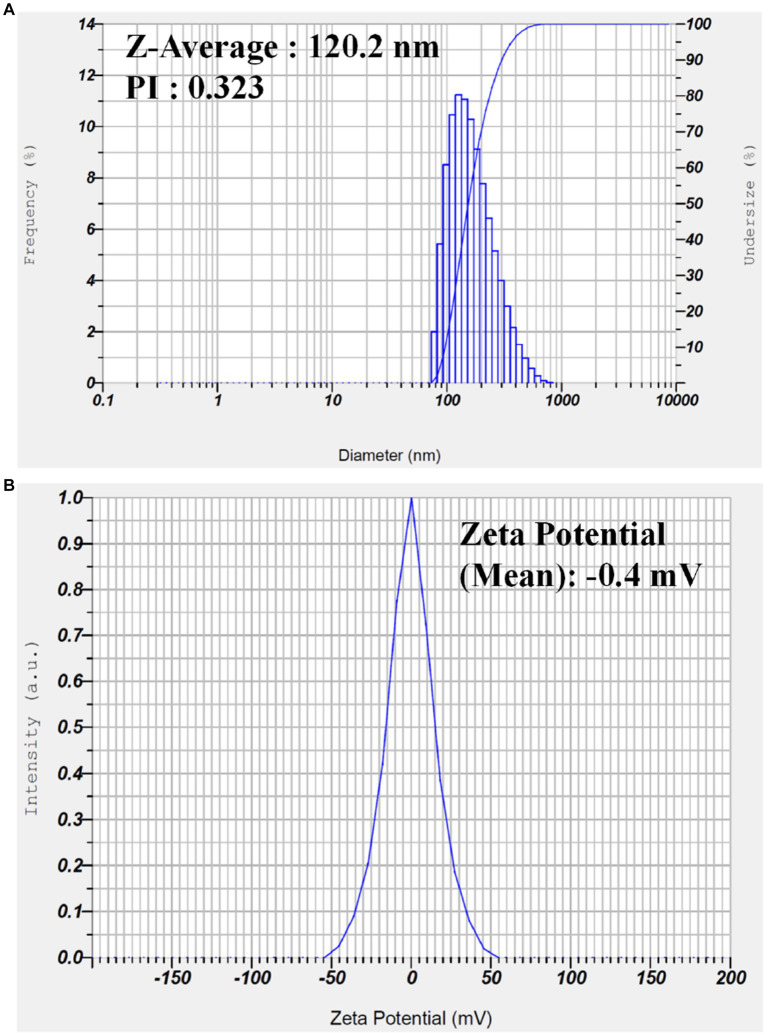
**(A)** Dynamic light scattering analysis to determine the particle size and **(B)** zeta potential analysis of the synthesized G-AgNP.

The size distribution and zeta potential of the G-AgNPs were determined by Dynamic Light Scattering Analysis (DLS), and obtained graphs were provided in Figure 6A,B. The particle size distribution indicates that G-AgNPs were monodispersed in nature, with an average diameter of ~120 nm. This range was not in accordance with the TEM results where particle size was observed between 20–30 nm, which might be due to the particle agglomeration effects in the solution phase. The mean polydispersity index (PdI) value of G-AgNPs was smaller than 0.4, indicating that G-AgNPs were distributed in monodisperse. The stability of G-AgNPs expressed through zeta potential with −0.4 mV. The negative potential value supports the high dispersity, good colloidal nature, and long-term stability of G-AgNPs.

EDAX analysis has been recorded to confirm the presence of elements and to confirm the impurities during the synthesis process. The obtained spectrum is provided in Supplementary Figure S2 (Supplementary information). Some elements identified in the G-AgNPs by EDAX are carbon, aluminum, and silver, with a weight percentage of 3.03, 2.54, and 94.43, respectively. The presence of aluminum in this nanoparticle was due to the prepared nanoparticles being coated over the aluminum foil to observe under the FESEM field. Hence this can be excluded from the elemental list in the prepared nanoparticles. Apart from this, high purity of the G-AgNPs was obtained without any impurities.

Typically, AgNPs play a vital role in antimicrobial activities, and colloidal silver was applied as a preservative agent in cosmetics (Sim et al., 2018). Several studies reported that AgNPs have been used in various biomedical assays, especially for bacterial infections, due to their effective antibacterial potential (Ahmad et al., 2020; Faisal et al., 2023). Silver’s colloidal formation efficiently acts as a preservative agent to develop shelf-life products (Liao et al., 2019; He et al., 2021). According to Lavanya et al. (2020), the gelatin compound stabilizes and reduces agents under temperature and pressure. In general, gelatin B polymer has a better surface negative charge coated with positive ions of AgNPs, and it becomes a better biocompatible agent in therapeutic applications (Van Vlierberghe et al., 2014). Thus, 4 mL of 0.2% colloidal G-AgNPs was used as an aqueous phase for the preparation of herb ointment.

In this study, the crude biosurfactant extracted from *P. aeruginosa* was used as an efficient emulsifier to reduce the blend between the oil and aqueous phases of the solution. Biosurfactants have better emulsifying capability depending on the value of Hydrophilic–Lipophilic balance (HLB) (Vecino et al., 2015). In addition, extracted glycolipid biosurfactants have excellent emulsification; therefore, it mainly considered for biomedical applications like dermatology.

### GCMS analysis of herb-infused oil

**Figure 7:**
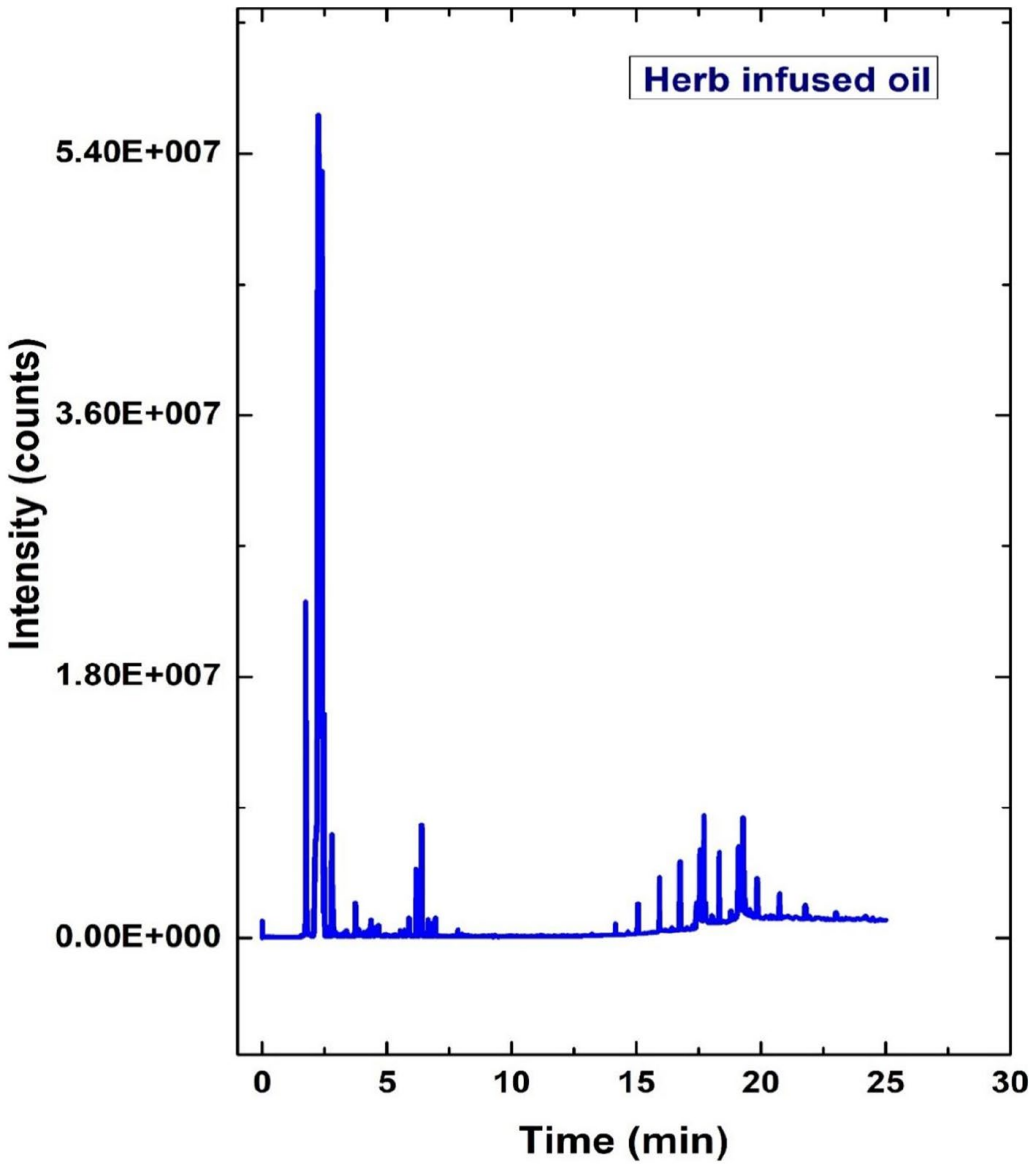
GCMS analysis of prepared herb-infused oil from *Tridax procumbens.*

The GCMS analysis evaluated the different peaks to identify the various compounds in the herb-infused oil, as illustrated in Figure 7. According to Figure 7, the other chemical compounds were identified, such as Ethane fluoro, Crinamidine, Octadecanoic acid, and Uracil, at the retention time of 1.33, 3.19, 6.57, and 14.97, respectively. Octadecanoic acid is most copious and present in the essential oil of plants like *Cynomorium songaricum* (Moussa and Almaghrabi, 2016). In general, octadecanoic acid is a common fatty acid used in food processing and cosmetics (Chen et al., 2019).

### Antimicrobial activity

**Figure 8:**
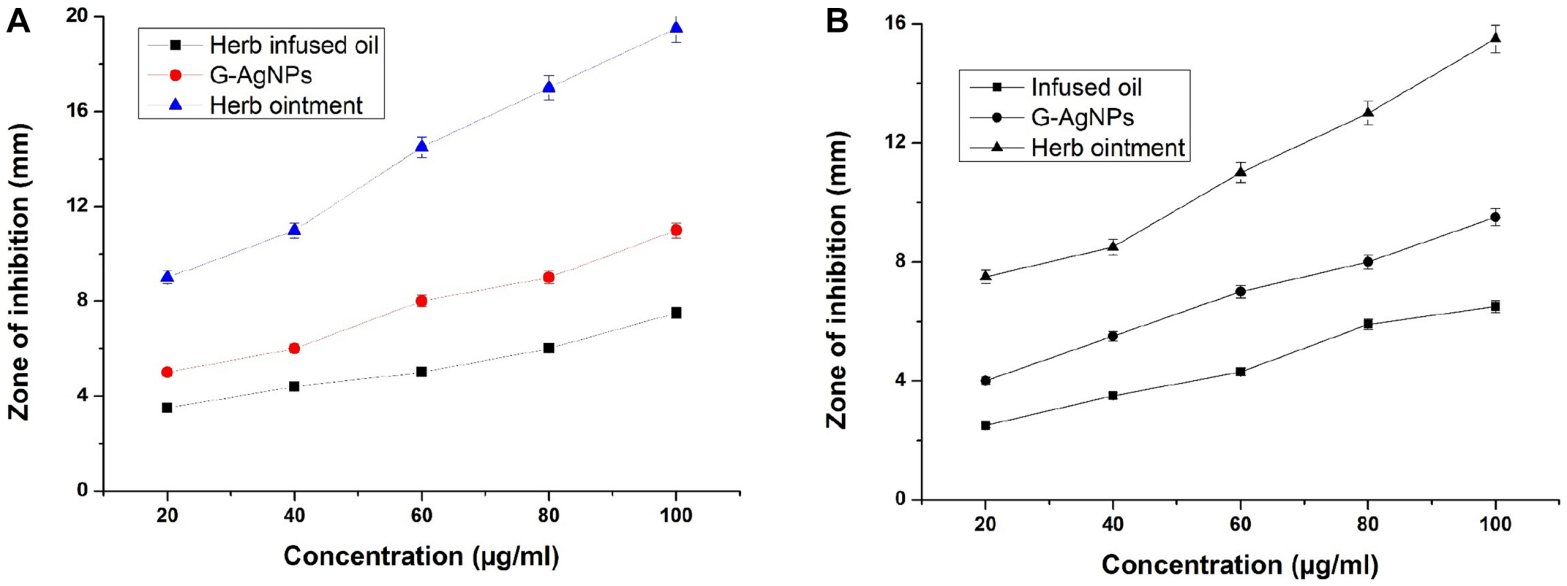
**(A)** Antibacterial activity of herb-infused oil, G-AgNPs, and herb ointment for *Staphylococcus aureus*. **(B)** Antibacterial activity for *E. coli*. The values are represented in ± SD Means (*p* < 0.05).

The prepared herb-infused oil, G-AgNPs, and herb ointment were examined for antimicrobial activities against two test organisms, *E. coli,* and *S. aureus,* with different concentrations (20–100 μg/mL) using Muller Hinton agar. The maximum inhibition of zone diameter was measured after the overnight incubation period. All experiments of antimicrobial activity results are shown in Figures 8A,B. This image shows herb ointment has the highest antibacterial activity compared to herb-infused oil and G-AgNPs. It shows the highest zone formation of 19.5 mm in the 100 μg/mL concentration against *S. aureus,* whereas 15.5 mm against *E. coli.* This could be possible by the effectiveness of potential secondary metabolites and G-AgNPs in the prepared herb ointment. The rhamnolipid biosurfactant molecule in the herb ointment also plays a better role in the antibacterial mechanism. This rhamnolipid biosurfactant causes cell disruption between the cell’s outer membrane and cytoskeletal elements by inserting the shorter acyl tails, lifting the membrane from the cytoplasmic constituents. Finally, it prevents bacterial activities by allowing toxic compounds (Bharali et al., 2013).

Similarly, infused oil shows the least inhibition of antibacterial activity at both pathogens compared to G-AgNPs and herb ointment at all concentrations. G-AgNPs showed better antibacterial activity over infused oil but could not achieve the level of herb ointment. The higher inhibition for G-AgNPs was 11 mm for *S. aureus* in 100 μg/mL (Figure 8A), whereas for *E. coli* found to be 9.5 mm (Figure 8B). Thus, the differentiation of antibacterial studies was observed clearly for infused oil, G-AgNPs, and herbal ointment against two clinical pathogens. The maximum zone inhibition area of prepared herb ointment from *Mangifera indica* extract was found to be about 11.8 mm for *E. coli* (El-Gied et al., 2015). As mentioned by Alalor et al. (2012), the ointment formulated using methanolic extracts of *Cassia alata* showed no growth reduction for *E. coli.* Similarly, Majumder and Majumder (2013) obtained a maximum inhibition of prepared herbal ointment from *Peperomia pellucida* extract was 12.5 mm for *E. coli.* Many studies reported that the extract of *Tridax* plant is a traditional plant with higher antibacterial properties due to saponins, tannins, alkaloids, and flavonoids (Thomas and McCubbin, 2003; Mundada and Shivhare, 2010). In a simple study, Christudas et al. (2012) reported that *T. procumbens* has significant antibacterial activity against bacterial strains *Bacillus faecalis, Bacillus subtilis* and *E. coli* in the range of 8–16 mm for the concentrations of 400–800 μg/mL. Similarly, the antioxidant, antibacterial, and cytotoxicity activity of leaves extract of *T. procumbens* were confirmed by Syed et al. (2020). In a recent study, Sophiaa et al. (2022) combined *T. procumbens* and *Acalypha indica* and found antibacterial activity against *E. coli* with 500 μg/mL. This mixed combination showed cytotoxicity activity against human breast cancer cells with the IC_50_ of 216.45 μg/mL. Compared to previously reported studies, our herbal ointment has better antibacterial activity against two test organisms, *E. coli* and *S. aureus.*

### Determination of MIC and MBC

In determining MIC, comparative efficacy of various concentrations (20, 40, 60, 80, and 100 μg/mL) of herb ointment were used. The results of the MIC study are shown in Table 1. From Table 1, 100 μg/mL of herb ointment shows better inhibition of *E. coli* and *S. aureus*. This result proved no visible growth in this concentration in both clinical bacteria. Low concentrations (20 and 40 μg/mL) of ointment results showed a higher turbidity solution in *E. coli* and *S. aureus.* Compared to this, only low turbidity solution was noted in the 60 and 80 μg/mL concentrations. These results revealed that the herb ointment has a better inhibition effect on *E. coli* and *S. aureus* growth. Several studies have reported that inhibition of the antibiotic Bacitracin with the preparation of topical ointment showed the MIC range at 256 μg/mL, and Mupirocin at 256–512 μg/mL against the pathogen *S. aureus* (Bonomo et al., 2007). As mentioned earlier, the concentration was higher than our prepared herb ointment to inhibit bacterial growth. In this study, the results of MBC always correspond to the MIC value, which demonstrates that the concentration of 100 μg/mL of herbal ointment killed the growth of two bacterial strains, namely *E. coli* and *S. aureus*, and confirmed it as bactericidal. As a result, the prepared herbal nano-infused ointment showed the more significant activities of MIC and MBC against selected pathogens.

**Table 1:** Minimum Inhibitory Concentration (MIC) of herb ointment at *Staphylococcus aureus* and *Escherichia coli*.

Concentration (μg/mL)	*Staphylococcus aureus*	*Escherichia coli*
control	–	–
20	0.9 ± 0.04	0.8 ± 0.03
40	0.8 ± 0.02	0.6 ± 0.02
60	0.6 ± 0.03	0.5 ± 0.04
80	0.4 ± 0.02	0.3 ± 0.02
100	0.06 ± 0.01	0.02 ± 0.01

### Cytotoxicity assay

**Figure 9:**
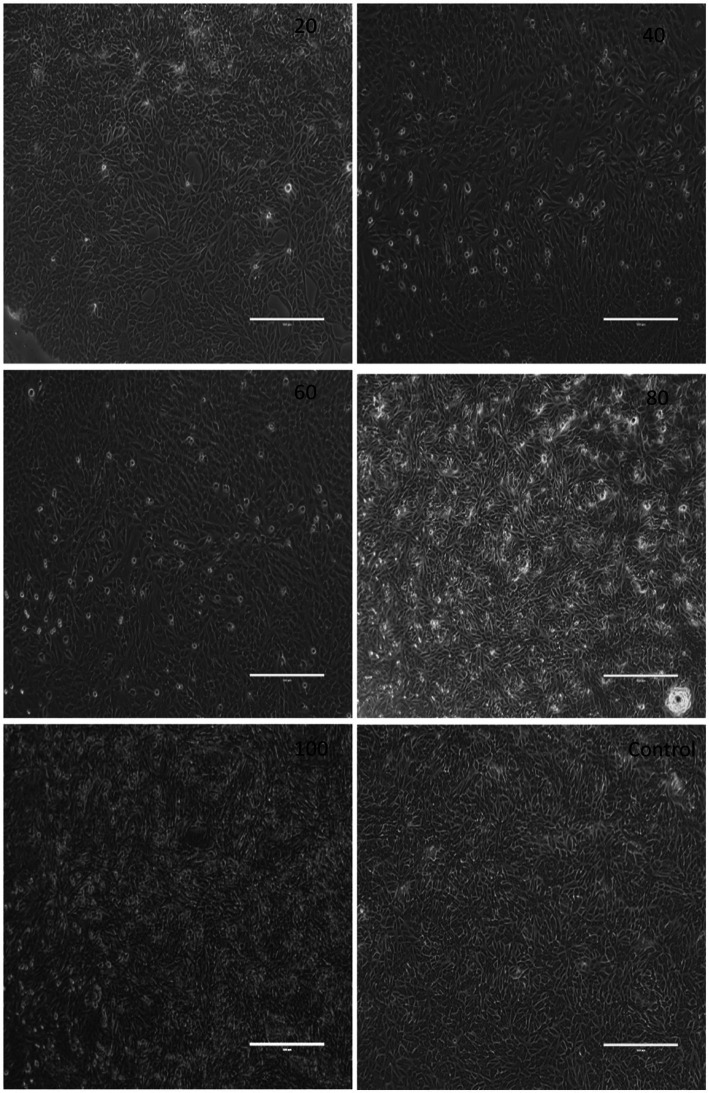
Digital photographic view of the cytotoxic effect of herb ointment on Vero cell lines at different concentrations.

The cytotoxic activity of herb ointment was tested against Vero normal cell line, and a significant difference was noted at different concentrations (20, 40, 60, 80, and 100 μg/mL), and the image was shown in Figure 9. The absorbance rate and cell viability percentage were shown in Table 2. From the table, the cell viability (%) of untreated cells (control) was 100%. Compared to untreated cells, a slight reduction was observed at different concentrations of herb ointment. The cell viability (%) of herb ointment at 20, 40, 60 80 and 100 μg/mL concentrations were found to be 99 ± 0.4%, 98 ± 0.5%, 95 ± 0.4%, 94 ± 0.7%, and 92 ± 0.6%, respectively. A slight cytotoxicity activity was noticed with increasing the concentration, but it was very less activity only. Machana et al. (2011) reported that the cytotoxic effect of the *Cladogynos orientalis* leaves extract was observed at about 38.7 ± 10.0 after 24 h treatment on Vero cell lines, whereas for *Catimbium speciosum* plant extract was observed at about 19.7 ± 2.5. This result shows that our nano-herb ointment has better cytotoxic activity on the Vero cell line. This result determined that the prepared herbal ointment could be applied in biomedical applications in the future.

**Table 2:** Absorbance rate and cell viability percentage of herb ointment.

Concentration (μg/mL)	Absorbance	Cell Viability %
Control	0.723 ± 0.003	100
20	0.718 ± 0.004	99 ± 0.4
40	0.712 ± 0.005	98 ± 0.5
60	0.694 ± 0.003	95 ± 0.4
80	0.684 ± 0.002	94 ± 0.7
100	0.665 ± 0.002	92 ± 0.6

### Wound scratch assay

**Figure 10:**
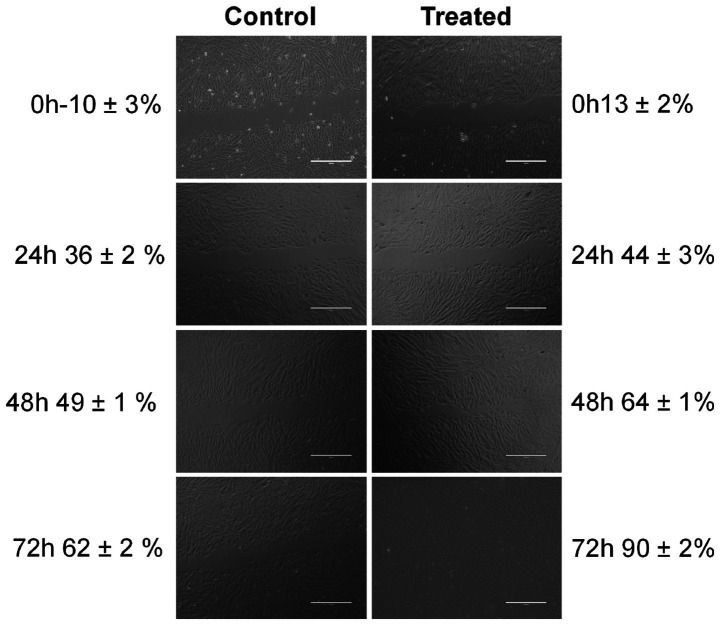
Photographic images of cell migration rate of herb ointment on wound healing at different time intervals.

The cell migration rate of the herb ointment on the Vero cell line was observed using a digital inverted microscope. The images of the wound scratch were monitored for control and treated cell line at 0, 24, 48, and 72 h during the incubation period. The percentage of cell migration was calculated in terms of the herb ointment. As shown in Figure 10, untreated cells showed only 10 ± 3% of cell migration at 0 h, whereas, in a treated cell line, 13 ± 2% of cell migration was obtained. Then continuously monitored in the different periods, the percentage of cell migration level in untreated cell lines at 24, 48, and 72 h was found to be 36 ± 2%, 49 ± 1%, and 62 ± 2%, respectively. Whereas in treated cell lines, the percentage of cell migration was found to be 44 ± 3%, 64 ± 1%, and 90 ± 2%, respectively. This result demonstrated that the rate of cell migration toward the created wound scratch area was accelerated in the presence of herb ointment. Moreover, the disappearance of wound areas showed the rapid occurrence of cell migration within 72 h (Figure 10). This could be influenced by the easy penetration of herb ointment on the infected wound site and stimulated the mechanism of inflammation, proliferation, and remodeling of the wound healing process. In this process, fibroblasts are mainly responsible for synthesizing collagen proteins on the wound site and forming the new capillaries by granulation (Midwood et al., 2004). Following that, the myofibroblasts made the wound contraction, decreased the closure size, and covered the edges by contracting mechanism (Gantwerker and Hom, 2012). In the epithelization process, new epithelial cells were grown by proliferation, covering the wound closure by forming the (Kirsner and Eaglstein, 1993; Sperandio et al., 2010). This assay revealed that the restoration of the tissues occurred rapidly on the wound site by the enhancement of epithelization.

Typically, dodecanoic, hexadecenoic, and octadecanoic acids have higher antimicrobial properties (Nitbani et al., 2022). Pu et al. (2010) reported that Octadecanoic acid has higher antibacterial properties against *S. aureus* and *E. coli* pathogens. Thus, the presence of dodecanoic acid and hexadecenoic acid in the extracted glycolipid biosurfactant and octadecanoic acid, which are present in the herb-infused oil, could play a vital to enhance the cell migration rate rapidly on the wound site by inhibiting the bacterial growth due to having higher antimicrobial properties. Many studies and clinical trials are tried to improve the wound-healing process. Nevertheless, in this study, prepared nano-based herbal ointment showed the complete re-epithelization of the wound area in 72 h incubation time. The results suggested that prepared ointment could be a potential biocompatible material and considered a more excellent wound healing agent.

## Conclusion

In this study, Herb Ointment (Biosurfactant/Herb infused oil/G-AgNPs) was prepared and characterized by FTIR and GCMS. GCMS results of the herbal-infused oil showed the presence of essential oil compounds. The FTIR spectra of biosurfactant showed the absorption peaks related to the hydroxyl group, aliphatic moieties, and carbohydrate compounds of rhamnose rings which correspond to the rhamnolipid biosurfactant. The MIC analysis shows better inhibition of *E. coli* and *S. aureus,* and the results confirmed the no visible growth in both bacteria at 100 μg/mL of herb ointment. Whereas, at low concentrations (20 and 40 μg/mL) ointment showed a higher turbid solution in both *E. coli* and *S. aureus.* Besides, the MBC results indicate that the 100 μg/mL concentration of herbal ointment killed both *E. coli* and *S. aureus*. The cytotoxic activity of herb ointment was studied using Vero normal cell line at different concentrations (20–100 μg/mL). The cytotoxicity assay results suggested that the herb ointment could be biocompatible for treating wounds at 100 μg/mL due to its better antibacterial properties. This study aims to evaluate the effectiveness of prepared nano-based herbal ointment on wound healing. The wound scratch assay confirms the cell migration rate of about 90 ± 2% in a 72 h incubation period than the control 62 ± 2%. This result revealed the complete disappearance of the scratch area within three days by the rapid occurrence of cell migration due to the herb ointment, which may stimulate epithelization, proliferation, and collagen viability around the wound region.

## Data availability statement

The raw data supporting the conclusions of this article will be made available by the authors, without undue reservation.

## Author contributions

MB: experimental work, field collection, and writing – original draft. PE: field collection and writing – review and editing. BM: data analysis, manuscript editing, and results and discussion. AS: experimental work and field collection. MA: resources, funding acquisition, and writing – review and editing. SD: validation, formal analysis, and results and discussion. PP: data analysis and analytical methods. AR: project administration, supervision, validation, and writing – review and editing. TM: validation and data analysis. MN: data analysis, manuscript editing, and results and discussion. All authors contributed to the article and approved the submitted version.

## Conflict of interest

The authors declare that the research was conducted in the absence of any commercial or financial relationships that could be construed as a potential conflict of interest.

PP and AR are guest editors of the submitted research topic.

## Publisher’s note

All claims expressed in this article are solely those of the authors and do not necessarily represent those of their affiliated organizations, or those of the publisher, the editors and the reviewers. Any product that may be evaluated in this article, or claim that may be made by its manufacturer, is not guaranteed or endorsed by the publisher.
