# Supplementary material for: Enhancement of cell migration and wound healing by nano-herb ointment formulated with biosurfactant, silver nanoparticles and Tridax procumbens
Source: Front Microbiol. 2023 Aug 2;14:1225769. doi: 10.3389/fmicb.2023.1225769 (PMC10434256; doi:10.3389/fmicb.2023.1225769)
Supplement: Supplementary file 1 [file Data_Sheet_1.docx]

**Supplementary Information**

**Enhancement of cell migration and wound healing by nano-herb ointment formulated with biosurfactant, silver nanoparticles and *Tridax procumbens***

Balakrishnan Muthukumar^1^, M.S. Nandini^2^, Punniyakotti Elumalai^3^, Balakrishnan Muthuraj^4^, Azhargarsamy Satheeshkumar^1^, Mohamad S. AlSalhi^5^, Sandhanasamy Devanesan^5^, Punniyakotti Parthipan^6***^, Aruliah Rajasekar^1* *^, Tabarak Malik^7*^

*^1^Environmental Molecular Microbiology Research Laboratory, Department of Biotechnology, Thiruvalluvar University, Serkkadu, Vellore, Tamil Nadu, 632115, India*

^2^*Department of Microbiology, Sree Balaji Medical College and Hospital, Chennai, Tamil Nadu, 600044 India.*

*^3^Green Laboratory, Microbiology and Environmental Toxicology Laboratory, Saveetha Institute of Medical and Technical Sciences, Saveetha Dental College, Poonamalle High Road, Chennai-600077, Tamilnadu, India.*

*^4^McKetta Department of Chemical Engineering, University of Texas at Austin, Austin, Texas, 78712, United States.*

*^5^Department of Physics and Astronomy, College of Science, King Saud University, P.O. Box-2455, Riyadh, 11451, Saudi Arabia*

*^6^Department of Biotechnology, Faculty of Science and Humanities, SRM Institute of Science and Technology, Kattankulathur, Chengalpattu, Tamil Nadu 603 203, India.*

*^7^Department of Biomedical Sciences, Institute of Health, Jimma University, Ethiopia.*

**Supplementary Figures**


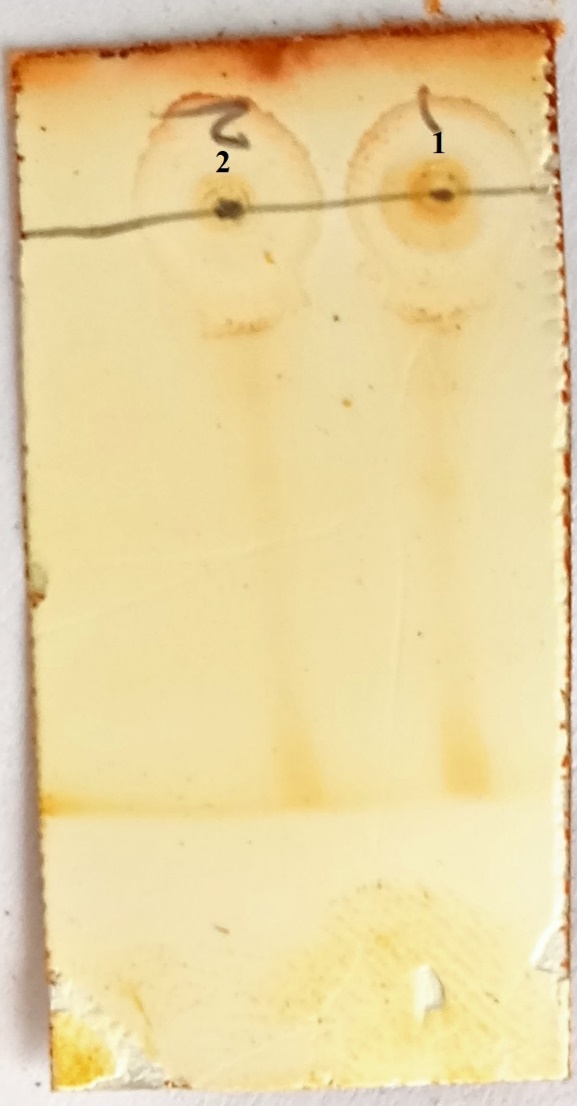


**Fig. S1.** Thin layer chromatography (TLC) of biosurfactant produced from strain *Pseudomonas aeruginosa* PP4 in lane 2; Lane 1 is standard rhamnolipid sample.

**Fig. S2.** EDAX spectrum of synthesized gelatin stabilized silver nanoparticle (G-AgNPs).
